# Plasma chaperone-mediated autophagy associated protein HSPA8 combined with white matter hyperintensities serves as the predictive marker of early-stage Alzheimer’s disease

**DOI:** 10.3389/fnagi.2026.1753692

**Published:** 2026-04-13

**Authors:** Xueqin Yan, Chunhua Liang, Yanxing Zhou, Guo Hong, Chengjie Li, Kaisuo Wang, Yaohui Huang, Xiaohua Xiao

**Affiliations:** 1 Department of Geriatric Medicine, The First Affiliated Hospital of Shenzhen University, Shenzhen Second People’s Hospital, Shenzhen, Guangdong, China; 2 Guangdong Key Laboratory for Biomedical Measurements and Ultrasound Imaging, National-Regional Key Technology Engineering Laboratory for Medical Ultrasound, School of Biomedical Engineering, Shenzhen University Medical School, Shenzhen, Guangdong, China

**Keywords:** Alzheimer’s disease, mild cognitive impairment, HSPA8 protein, white matter hyperintensities, early diagnosis

## Abstract

**Background:**

The relationship between plasma chaperone-related autophagy proteins and white matter hyperintensity (WMH) in Alzheimer’s disease (AD) remains unclear.

**Methods:**

We employed 4D-DIA proteomics to identify plasma protein changes, and evaluated the clinical relevance of the chaperone-mediated autophagy (CMA)-related protein HSPA8. Additionally, using ITK-SNAP software to assess WMH volume’s role in AD. We analyzed the ROC curves for both HSPA8 and WMH in the AD spectrum. Among which, using One-Anova, Kruskal–Wallis, and multivariable logistic analyses to detect the population data. Moreover, the impact of age on WMH volume changes between the AD group and the CN group will be assessed using sensitivity analysis and testing the age-diagnosis interaction.

**Results:**

Significant factors in the AD population included age, MMSE score, MoCA score, and WMH volumes. The OR for age in MCI and for WMH volume in AD patients were significant. The expression of HSPA8 was decline in the AD disease spectrum, but it had no statistical difference. Importantly, HSPA8 protein had the highest AUC value for distinguishing between cognitively normal (CN)/mild cognitive impairment (MCI) and MCI/AD groups. Meanwhile, WMH was significant in the AD disease spectrum. And the influence of age on WMH is comparable in cognitively normal elderly individuals and those with AD. Combining HSPA8 with WMH improved the AUC value, which was further enhanced by including age and gender.

**Conclusion:**

We found that the level of CMA-related protein HSPA8 is decline in the AD disease continuum, and WMH volume may help differentiate the AD spectrum. Moreover, Age is the primary factor influencing WMH changes in both CN and AD groups, with AD status having no significant effect on WMH levels or their progression. Thus, age should be carefully considered when evaluating elevated WMH in AD patients. It is noteworthy that the integration of HSPA8 and WMH may function as a potential early biomarker for AD, thereby enhancing the accuracy of its early diagnosis. Including age and gender increases the diagnostic model’s AUC value, indicating HSPA8 and WMH are crucial for early AD diagnosis.

## 1 Introduction

Alzheimer’s disease (AD) is a leading cause of dementia in older adults, characterized by β-amyloid plaques and neurofibrillary tangles. By 2050, it is expected to affect over 152 million people globally (GBD 2019 Dementia Forecasting Collaborators, 2022), with China’s cases rising from 10 to 48 million (GBD 2016 Dementia Collaborators, 2019; Jia et al., 2020). AD presents a major personal and economic challenge worldwide, with no effective treatment to stop its progression (Panza et al., 2019). Early diagnosis is difficult, but advances in blood-based biomarkers offer a promising, reliable, non-invasive, and cost-effective solution (Molinuevo et al., 2018). High-sensitivity assays have proven valuable for AD screening and diagnosis. Identifying molecular markers is essential for early detection and better treatment outcomes (Kim et al., 2023), and blood-based biomarkers can help identify high-risk populations (Teunissen et al., 2022).

Autophagy is a process of recycling intracellular proteins and aging organelles, occurring under certain conditions (Parzych and Klionsky, 2014). It is linked to the progression of AD and plays a key role in various disorders, including neurodegenerative diseases, inflammation, cardiovascular disorders, cancer, and aging (Levine and Kroemer, 2019). In mammals, autophagy is categorized into macroautophagy, microautophagy, and chaperone-mediated autophagy (CMA), with macroautophagy being crucial for clearing amyloid-beta deposits in AD (Li et al., 2017; Zamani et al., 2019). A study identified nine blood genes (ATG16L2, BAK1, CAPN10, CASP1, RAB24, RGS19, RPS6KB1, ULK2, and WDFY3) related to autophagy as potential AD biomarkers (Qin et al., 2022). Veverová et al. (2024) and Veverová et al. (2025) studied biomarkers related to autophagy and lysosomal biogenesis (PINK1, ULK1, BNIP3L, and TFEB) in biofluids [cerebrospinal fluid (CSF) and serum] across different cognitive conditions (the frontotemporal lobar degeneration continuum, AD continuum, and cognitively unimpaired controls). The macroautophagy–lysosomal system and CMA are key autophagic pathways in AD (Hein et al., 2025). Thus, identifying blood proteins related to CMA could help detect early AD.

In addition to Aβ plaques and tau tangles, biomarkers of vascular damage and inflammation are crucial in AD. Studies have linked specific blood protein expressions to AD, mild cognitive impairment (MCI) (Pase et al., 2019), brain MRI, amyloid-PET burden (Shi et al., 2019), and cognitive decline (Tanaka et al., 2020). Many individuals in China with MCI or dementia also have cerebrovascular disease (Jia et al., 2020). The updated diagnostic criteria now include inflammation, vascular brain injury, and α-synuclein, with white matter hyperintensities (WMHs) as a vascular biomarker (Jack et al., 2024). WMHs can indicate AD severity and progression (Alosco et al., 2018). A study found that plasma neurofilament light chain (NfL) is significantly associated with WMHs in a U.S. cohort (Vítor et al., 2023). While plasma biomarkers can indicate neuroimaging changes in AD, the combined impact of AD biomarkers and vascular burden on prognosis in memory clinic patients is still unclear (van Gils et al., 2023).

The complex nature of AD underscores the need for multiple blood biomarkers for accurate prediction. Plasma proteomics can uncover new biomarkers related to AD mechanisms like neuroinflammation and oxidative stress (Zhang et al., 2024). Gomez et al. (2024) identified 13 plasma proteins associated with WMH linked to synaptic function and angiogenesis, offering insights into potential AD biomarkers. This study seeks to discover plasma biomarkers associated with other AD mechanisms and WMH changes in individuals with mild cognitive impairment (MCI) and AD.

Emerging proteomic technologies, such as 4D-DIA mass spectrometry, enhance Alzheimer’s research by providing highly sensitive and precise protein detection (Guo and Aebersold, 2023).

Unlike traditional 3D proteomics, 4D-DIA incorporates ion mobility, boosting scanning speed and sensitivity while ensuring nearly complete precursor ion acquisition (Meier et al., 2020), thereby reducing the likelihood of missing low-abundance proteins (Aebersold and Mann, 2016). Using plasma 4D-DIA proteomics, we discovered a new protein and, along with WMH, classified the AD disease spectrum.

## 2 Materials and methods

### 2.1 Study cohort

Subjects were recruited from the First Affiliated Hospital of Shenzhen University from January 2023 to December 2023. AD inclusion criteria were: (1) aged 50–85; (2) latent onset with early subjective cognitive decline and slow progression; (3) neuropsychological assessment scores: MMSE (Folstein et al., 1975; Katzman et al., 1988) (illiteracy ≤ 17, primary school ≤ 20, junior high and above ≤ 24) or MoCA-B (Lu et al., 2011) (illiteracy and primary school ≤ 19, secondary school ≤ 22, university ≤ 24).

MCI criteria included: (1) aged 50–85; (2) bjective cognitive decline evident in tests or compared to prior performance, affecting areas like memory, language, attention, or executive function, but not severe enough to disrupt daily life or social functions, and not meeting dementia diagnostic criteria. (3) Objective examinations indicate MCI, with MMSE scores of 18–21 for illiterates, 21–24 for primary education, and 25–27 for secondary education. (4) The disease duration exceeds 3 months.

Cognitively normal (CN) individuals are (1) aged 50–85; (2) have MMSE and MoCA scores ≥ 27; (3) report no cognitive decline; (4) have no neuropsychiatric diagnoses; (5) possess adequate communication skills for cognitive tests; and (6) demonstrate normal daily living abilities.

Exclusion criteria include (1) major medical, neurological, or psychiatric conditions aside from AD; (2) substance abuse; (3) delirium; (4) significant cerebrovascular history; (5) Hachinski Ischemic Score > 4; (6) severe sensory impairments; and (7) inability to complete neuropsychological assessments or poor-quality MRI scans.

Patients with MCI fulfilled the 2011 diagnostic criteria issued by the National Institute on Aging (NIA) and the Alzheimer’s Association (AA), which define AD-derived MCI; patients with AD meet the 2011 NIA–AA diagnostic criteria for probable AD dementia and probable AD dementia (McKhann et al., 2011).

Demographic features considered in the analysis included age, sex, hypertension and diabetes mellitus. Age was analyzed as continuous variables.

### 2.2 Collecting blood

The venous blood (∼10 mL) samples were collected by purple top EDTA tubes. plasma was obtained by removing blood cell debris using centrifugation at 3,000 g for 10 min at 4 °C. The plasma samples were then stored at—80 °C for storage.

### 2.3 Plasma 4D-DIA proteomics analysis

Fifty nine plasma samples of Alzheimer’s disease were analyzed using 4D-DIA proteomics. High and low abundance proteins were separated with the Human 14 Multiple Affinity Removal System (Agilent Technologies) and concentrated using a 5 kDa ultrafiltration tube (Sartorius). For DDA Mass Spectrometry, samples were analyzed with a TIMSTOF mass spectrometer (Bruker) via Evosep One liquid chromatography (Denmark) in data-dependent mode to create a spectral library. For DIA Mass Spectrometry, peptides were analyzed in DIA mode using the same equipment. DDA library data was searched with Spectronaut*™* software, and DIA data was analyzed using the constructed spectral library. The software was configured with dynamic indexed retention time prediction, MS2 level interference correction, and cross-run normalization. Results were filtered with a Q value cutoff of 0.01 (FDR < 1%). The 4D-DIA proteomic analysis was conducted by Suzhou PANOMIX Biomedical Tech Co., Ltd, China.

### 2.4 The WMH volume detection

All participants underwent head examination using German Siemens Prisma 3.0 T or Avanto 1.5 T magnetic resonance scanner with scanning layer thickness as 6 mm. The scan sequences included T1-weighted imaging (TlWI), T2-weighted imaging (T2WI) and fluid-attenuated inversion recovery (FLAIR), etc. Total WMH were evaluated by the physician without being informed of the patient’s basic condition and cognitive function. With WMH as Region of Interest (ROI), T1WI, T2WI, and FLAIR images of head MRI were interpreted by two professionally trained physicians, and WMH regions were manually delineated using ITK-SNAP software. The WMH manual sketching results are then reviewed and modified by senior physicians to ensure the accuracy of the results.

### 2.5 Statistical methods

The SPSS software (version 27) was used for data analysis. One-way ANOVA was used for the quantitative data that conformed to a normal distribution, and the Kruskal–Wallis test was used to compare data skewness. Multivariable logistic analysis to test the beta coefficient and OR of age, sex, hypertension, diabetes mellitus, HSPA8 and WMH volume in AD disease. Numerical variables of clinical profiles were shown as mean values ± SD or median (inter-quartile range). The receiver operating characteristic (ROC) curves were generated to quantify the predictive accuracy of the models, and the area under the receiver operating characteristic curve (AUC) was used to assess the discriminatory ability of the models. The Spearman correlation was used to measure the degree of association between the discovered markers and AD assessment scores. To control for age and sex confounding effects on WMH, we conducted a sensitivity analysis was performed using a subsample (*n* = 6) from the CN cohort, matched to the AD group by age and sex, to compare WMH differences using either an independent-samples *t*-test. Then, we tested the age-by-diagnosis interaction using a multiple linear regression model with WMH as the dependent variable and age, diagnostic group, and their interaction as independent variables. A significant interaction (*P* < 0.05) would indicate differing age effects on WMH between groups. One-way ANOVA, the Kruskal–Wallis test and multivariable logistic analysis were performed using SPSS software. The ROC curves, the Spearman correlation, and the plot of HSPA8 protein and WMH volumes in AD disease spectrum were performed using Graphpad prism (version 9.0).

## 3 Results

We studied plasma proteomics and WMH volume in 59 participants: 15 with AD, 22 with MCI, and 22 CN controls. Table 1 details their demographics and biomarkers. Age, MMSE, MoCA, and WMH volume were significant across the AD spectrum via One-Way ANOVA or Kruskal–Wallis tests. Plasma HSPA8 protein levels were 80.27 [45.79, 99.46] in CN, 78.63 [63.09, 88.62] in MCI, and 60.40 [39.70, 94.13] in AD, showing no significance. Average ages were 62.73 (CN), 71.27 (MCI), and 80.13 (AD) years, with women making up 59.1, 54.5, and 46.7% of each group. Hypertension rates were 40.9% (CN), 59.1% (MCI), and 53.3% (AD), while diabetes rates were 31.8% (CN), 36.4% (MCI), and 20.0% (AD). Median MMSE scores were 27 (CN), 26 (MCI), and 19 (AD), and MoCA scores were 26 (CN), 22 (MCI), and 18 (AD). The WMH volume was 1688 (CN), 3005 (MCI), and 18549 (AD). Multivariate logistic analysis for age, sex, hypertension, diabetes, HSPA8, and WMH is shown in Table 2, The results indicated the following beta coefficients: for MCI, they were –0.216, –1.242, –0.196, 1.580, –0.025, and –0.0001; for AD, they were –0.141, –0.291, 1.089, 1.033, –0.008, and –0.0001. The odds ratio (OR) for the age of MCI patients was 0.805 [95% CI 0.688–0.943, *P* = 0.007], and for the WMH volume of AD patients, it was 1.000 [95% CI 1.000–1.000, *P* = 0.044]. Other variates did not show significant ORs.

**TABLE 1:** Demographic and clinical characteristics of the study participants.

Groups	CN (*n* = 22)	MCI (*n* = 22)	AD (*n* = 15)	*P*
Age, Y	62.73 ± 9.78	71.27 ± 9.21	80.13 ± 7.00^a,b,c^	<0.001
Female (%)	13(59.1%)	12(54.5%)	7(46.7%)	0.767
Hypertension (%)	9 (40.9%)	13 (59.1%)	8 (53.3%)	0.197
Diabetes mellitus (%)	7 (31.8%)	8 (36.4%)	3 (20.0%)	0.207
MMSE score	27.00 [26.00, 28.25]	26.00 [23.50, 28.00]	19.00 [16.25, 22.50]^a,c^	<0.001
MoCA score	26.00 [25.00, 27.00]	22.00 [18.50, 24.50]	18.00 [11.50, 20.00]^a,c^	<0.001
plasma HSPA8 (absolute peak area)	80.27 [45.79, 99.46]	78.63 [63.09, 88.62]	60.40 [39.70, 94.13]	0.248
WMH volume (mm^3^)	1668.00 [683.90, 5645.00]	3005.00 [1210.00, 8678.00]	18549.00 [6771, 35053]^a,b^	0.002

For continuous variables, data are shown as mean (standard deviation) or median [inter-quartile range]; for categorical variables, data are given as absolute numbers (proportions). *P*-values are derived from One-Anova and Kruskal–Wallis tests. CN, MCI, AD: Cognitively normal, mild cognitive impairment, and Alzheimer’s disease, respectively; MMSE, Mini-Mental State Examination; MoCA, Montreal Cognitive Assessment; HSPA8, Heat shock protein family A member 8; WMH, White matter hyperintensities;

*^a^*CU vs. MCI (*P* < 0.05),

*^b^*CU vs. AD (*P* < 0.05),

*^c^*MCI vs. AD (*P* < 0.05).

**TABLE 2:** The beta coefficient, OR and OR CI of multivariable to MCI or AD patients.

	MCI				AD			
Groups	Beta	OR	OR CI	*P*-value	Beta	OR	OR CI	*P*-value
Intercept	19.881				12.251			
Age	–0.216	0.805	0.688–0.943	0.007	–0.141	0.869	0.754–1.002	0.053
Sex	–1.242	0.289	0.021–4.035	0.893	–0.291	0.748	0.062–9.069	0.819
Hypertension	–0.196	0.822	0.047–14.225	0.389	1.089	2.970	0.218–40.426	0.414
Diabetes mellitus	1.580	4.857	0.133–177.685	0.053	1.033	2.811	0.117–67.453	0.524
Plasma HSPA8	-0.025	0.975	0.951–1.000	0.060	–0.008	0.992	0.974–1.010	0.390
WMH volume	–0.0001	1.000	1.000–1.000	0.356	–0.0001	1.000	1.000–1.000	0.044

OR, odd ratio; CI, confidence internal.

### 3.1 The different expression proteins of plasma by 4D-DIA proteomics

**FIGURE 1:**
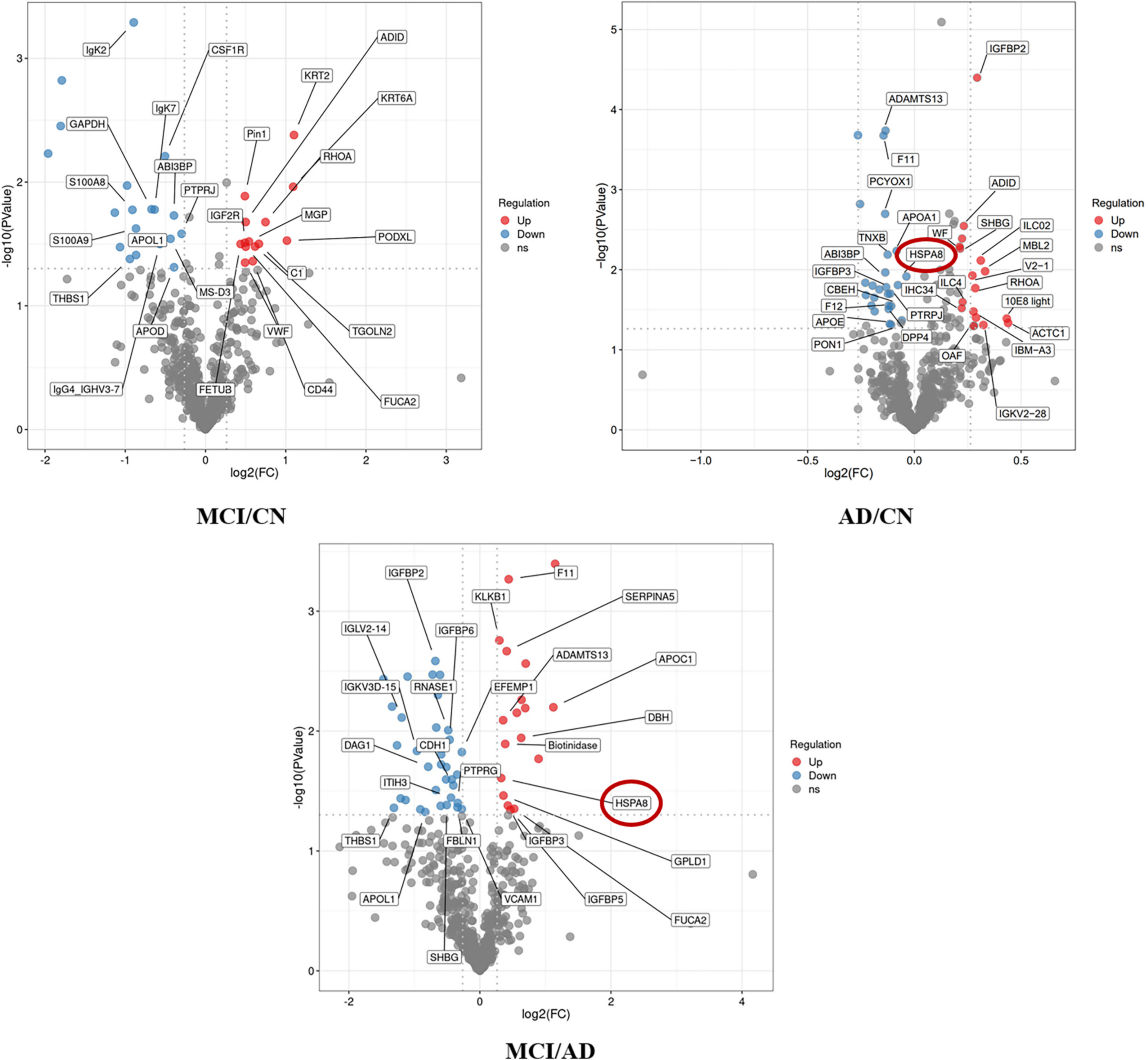
The volcano plot of differentially expression proteins in the AD disease spectrum. The horizontal axis shows the fold change (log_2_), and the vertical axis shows the significance *P*-value (log_2_). Red dots indicate up-regulated proteins, blue dots indicate down-regulated proteins, and gray dots indicate unchanged proteins. Points with IDs represent the top 20 differentially expressed proteins.

**FIGURE 2:**
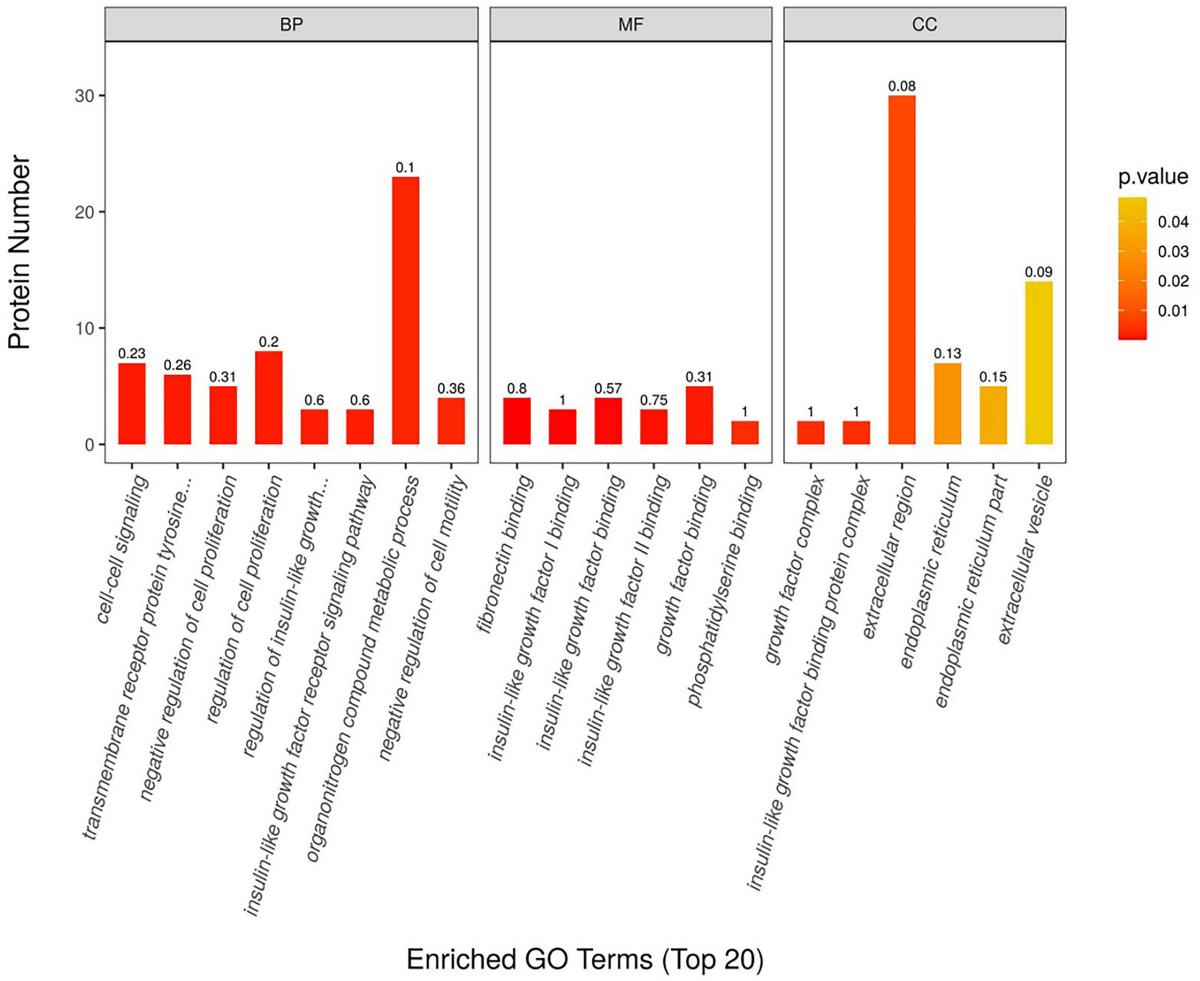
The result of the GO function in the MCI vs. AD group. The figure’s vertical axis shows GO level functional annotations—biological process, molecular function, and cellular component—to differentiate blue, red, and orange. The horizontal axis indicates the protein count for each functional classification, with the enrichment factor (Rich Factor ≤ 1).

**FIGURE 3:**
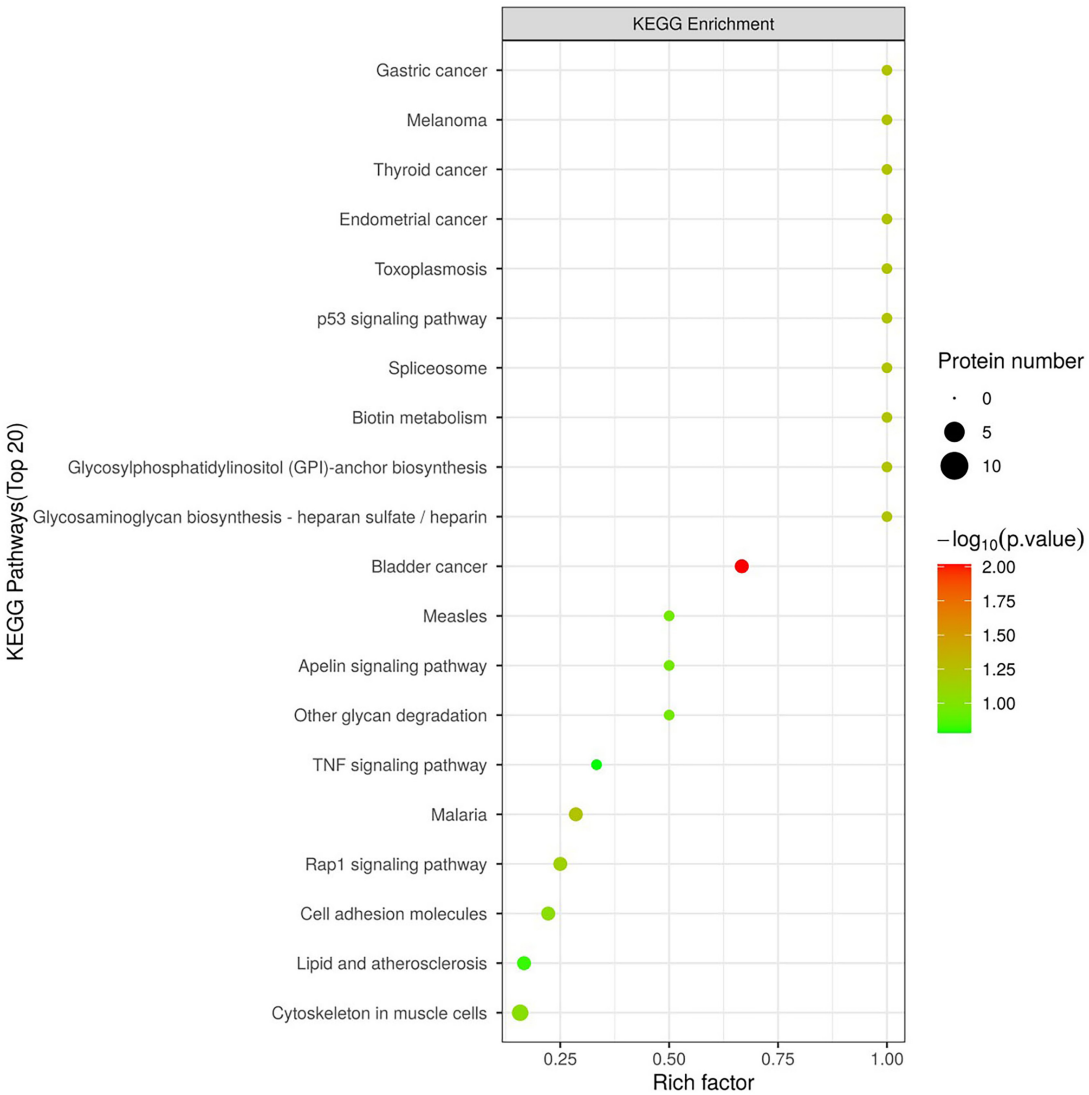
The KEGG of differentially expression proteins in the MCI vs. AD group. The horizontal coordinate represents the enrichment factor (Rich Factor ≤ 1), indicating the ratio of differentially expressed proteins to all identified proteins in a KEGG pathway. Bubble size shows the number of different proteins per pathway, while bubble color reflects enrichment significance, with a color gradient indicating the *P*-value size (-log_10_). Redder colors signify smaller *P*-values and higher enrichment significance.

We used 4D-DIA proteomics to analyze plasma proteins in individuals with CN, MCI, and AD, identifying 894 proteins across the AD continuum. We highlighted the top 15 differentially expressed proteins in the MCI/AD group, including insulin-like growth factor binding proteins (IGFBP), HSPA8, Biotinidases, and S100A8/S100A9. Notably, HSPA8 was present in both the MCI/AD and AD/CN groups, showing down-regulation alongside IGFBP3, Glycosyl-phosphatidylinositol specific phospholipase D1 (GPLD1), and Biotinidase in the MCI vs. AD comparison (Figure 1). Gene Ontology analysis indicated involvement in the insulin-like growth factor receptor signaling pathway (Figure 2), while KEGG pathway enrichment identified the p53 signaling pathway, biotin metabolism, and TNF pathway among the top 20 pathways (Figure 3).

### 3.2 Plasma candidate proteins associated with the disease spectrum of AD

**FIGURE 4:**
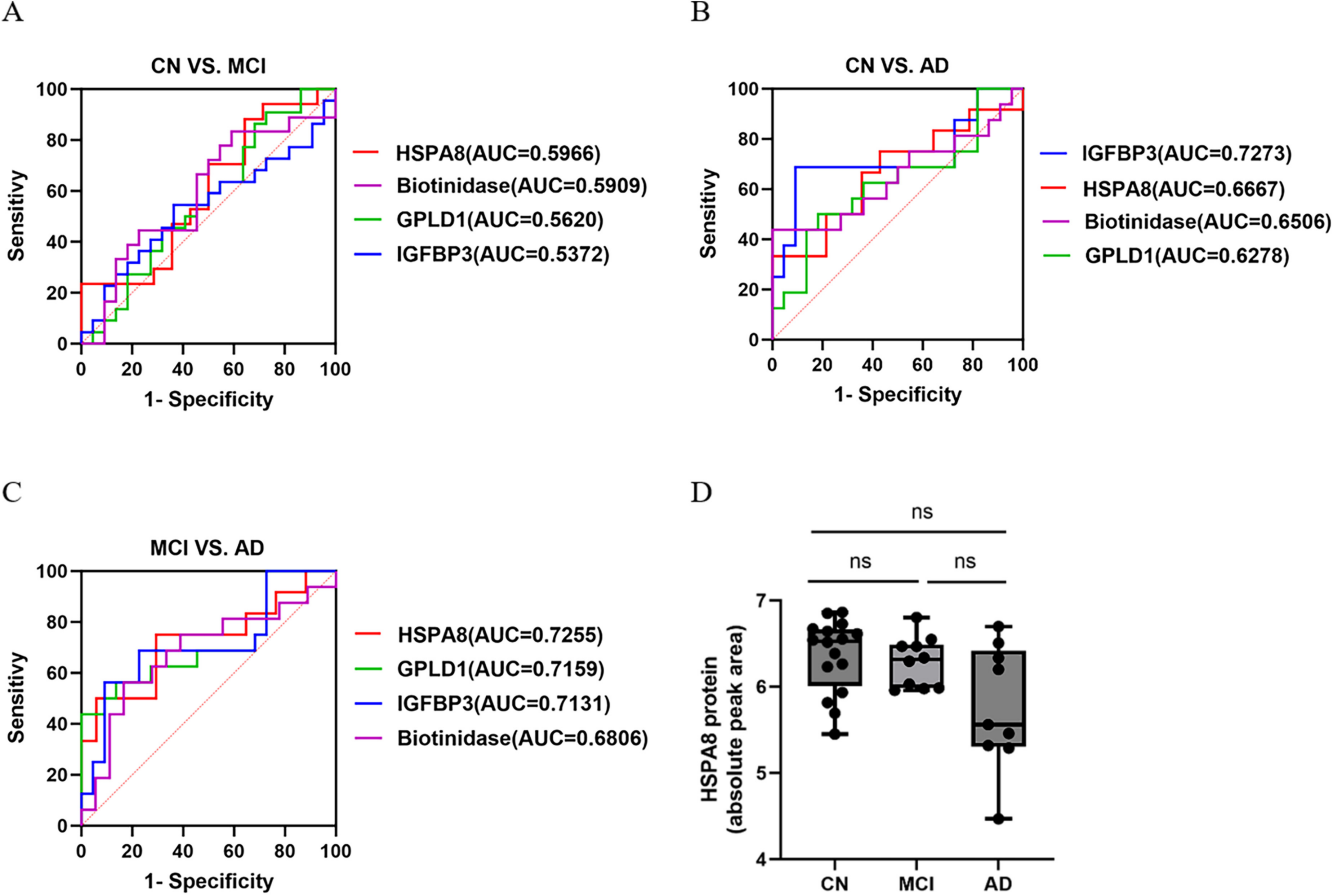
The AUC value of candidated four proteins to the AD disease continuum. **(A)** The ROC curve of HSPA8, Biotinidase, GPLD1 and IGFBP3 to CN vs. MCI. **(B)** The ROC curve of HSPA8, Biotinidase, GPLD1 and IGFBP3 to CN vs. AD. **(C)** The ROC curve of HSPA8, Biotinidase, GPLD1, and IGFBP3 to MCI vs. AD. **(D)** The expression of HSPA8 protein in the AD disease spectrum. One-way ANOVA analyzed the differences in HSPA8 protein expression among the CU, MCI, and AD groups. ns indicates a *P*-value > 0.05 for CN vs. MCI, AD vs. CN and AD vs. MCI. The absolute peak area of HSPA8 protein was indicated as log_2_.

**FIGURE 5:**
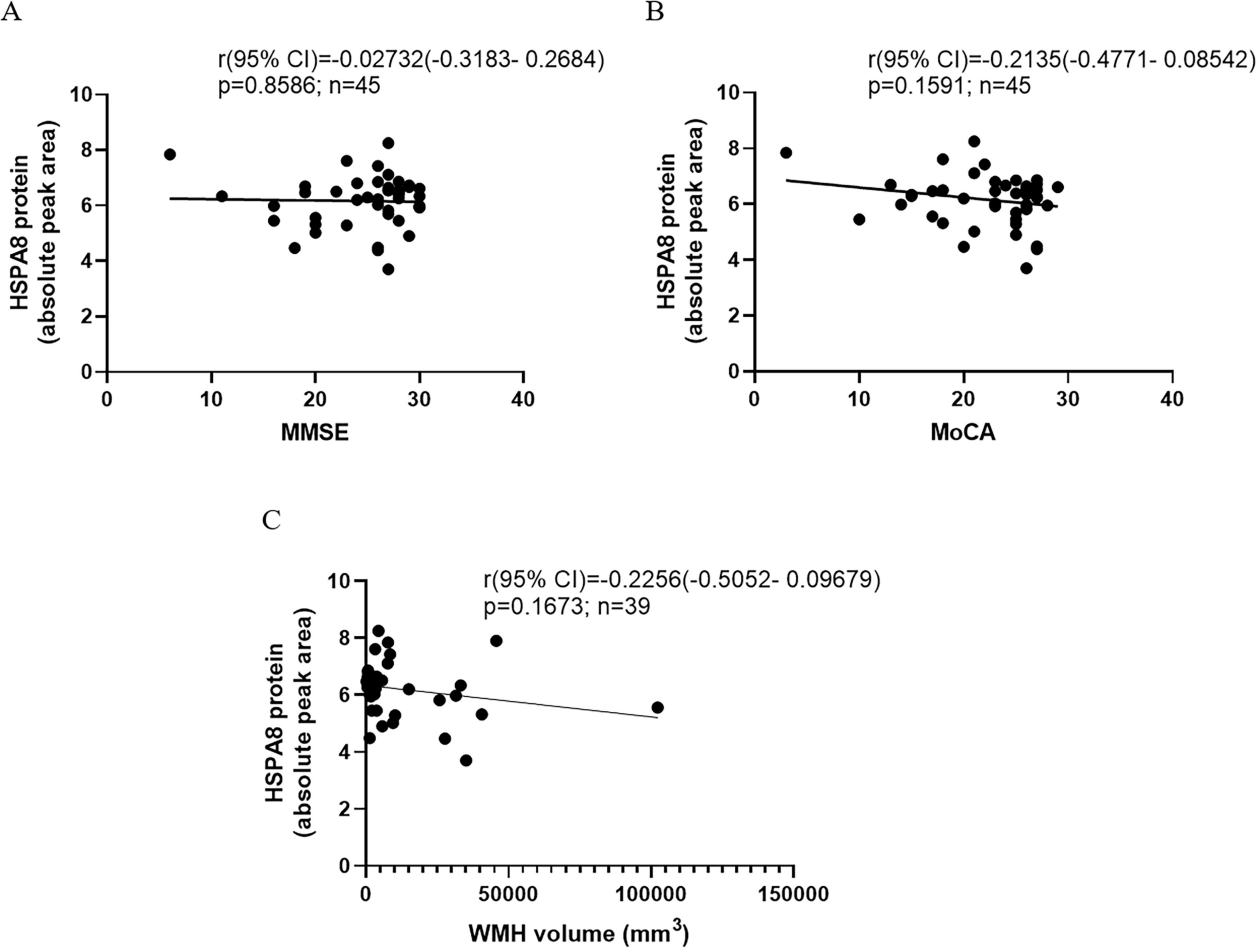
The correlation between HSPA8 protein and cognitive scores or the WMH volume. **(A)** The correlation between HSPA8 and MMSE. **(B)** The correlation between HSPA8 and MOcA. **(C)** The correlation between HSPA8 and WMH.

The AUC values for distinguishing MCI from controls were: HSPA8 at 0.5966, GPLD1 at 0.5620, IGFBP3 at 0.5372, and Biotinidase at 0.5909. For AD vs. controls, the AUCs were: HSPA8 at 0.6667, GPLD1 at 0.6278, IGFBP3 at 0.7273, and Biotinidase at 0.6506. In MCI vs. AD, the AUCs were: HSPA8 at 0.7255, GPLD1 at 0.7159, IGFBP3 at 0.7131, and Biotinidase at 0.6806 (Figures 4A–C). HSPA8 showed the highest AUC in both CN/MCI and MCI/AD groups, warranting further analysis. Importantly, we showed the decline of HSPA8 protein in the AD disease continuum, but there was not statistically significant (Figure 4D). The Pearson correlation coefficients for HSPA8 levels with cognitive scores were: MMSE at –0.02732 (*P* = 0.8586), MoCA at –0.2135 (*P* = 0.1591), and WMH at –0.2256 (*P* = 0.1673) (Figure 5).

### 3.3 The role of WMH volume in MCI and AD patients

**FIGURE 6:**
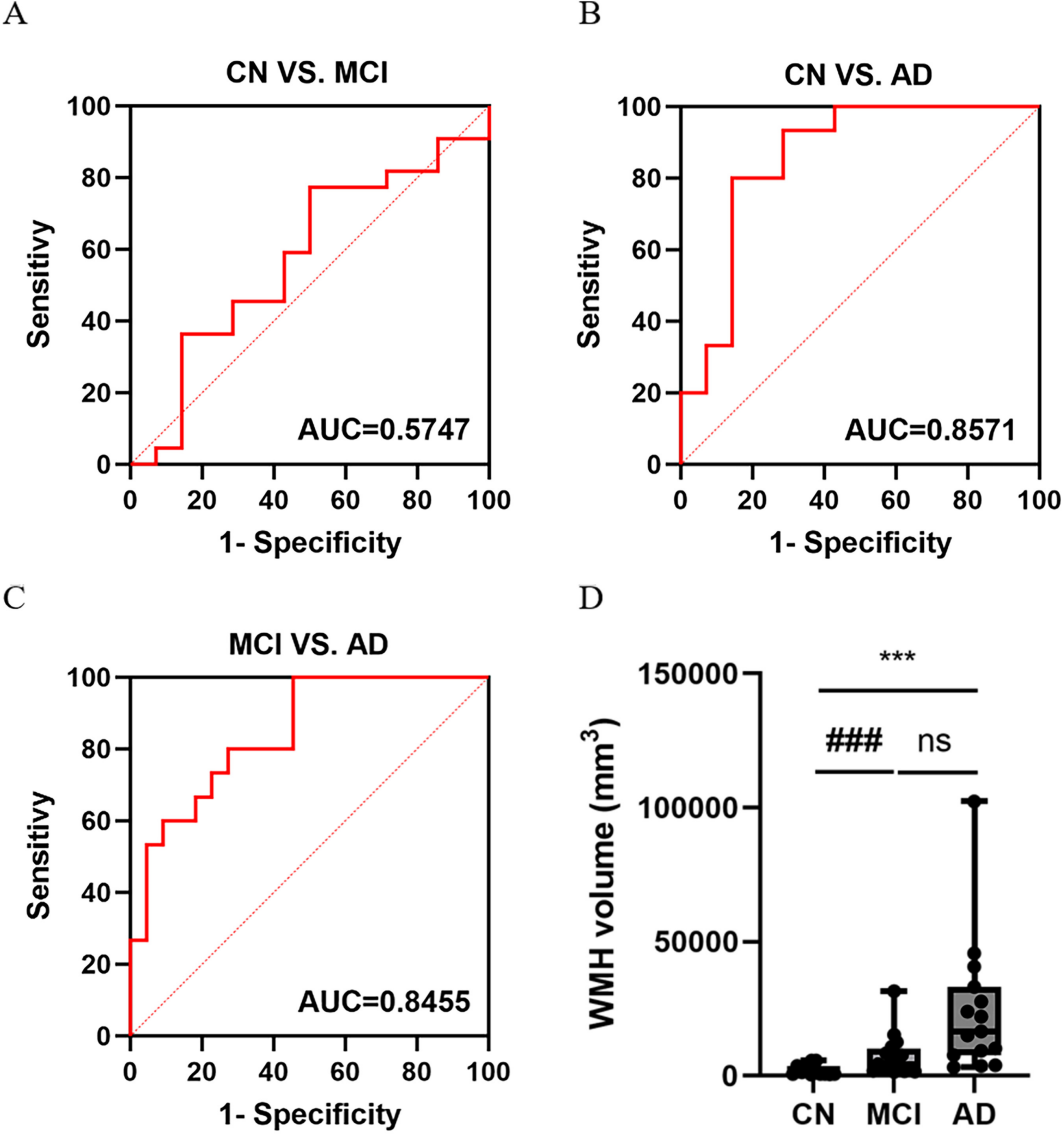
The AUC value of WMH to the AD disease continuum, and the expression of WMH in the AD disease spectrum. **(A)** The ROC curve of WMH to CN vs. MCI. **(B)** The ROC curve of WMH to CN vs. AD. **(C)** The ROC curve of WMH to MCI vs. AD. **(D)** The volume of WMH in the AD disease spectrum. One-way ANOVA analyzed the differences in WMH volume among the CU, MCI and AD groups. ns indicates a *P*-value > 0.05 for MCI vs. AD; *** indicates a *P*-value < 0.01 for AD vs. CN; ### indicates a *P*-value < 0.01 for MCI vs. CN.

**FIGURE 7:**
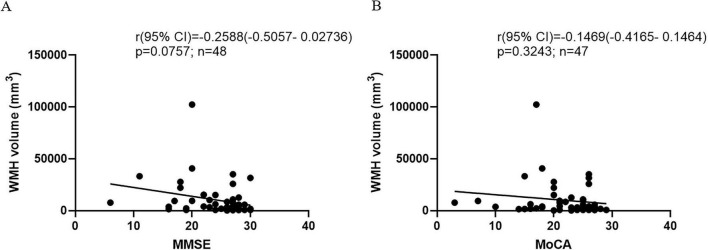
The correlation between WMH and cognitive scores. **(A)** The correlation between WMH and MMSE. **(B)** The correlation between WMH and MoCA.

The AUC values of WMH volume were 0.5747 for MCI vs. CN, 0.8571 for AD vs. CN, and 0.8455 for MCI vs. AD. WMH volume increased across the AD spectrum (Figure 6). The Pearson correlation between WMH volume and cognitive scores was -0.2588 (*P* = 0.0757) for MMSE and -0.1469 (*P* = 0.3243) for MoCA (Figure 7). In the multivariate regression analysis, age emerged as a variable of significant distinction. Furthermore, within the demographic data of the population, age holds considerable significance. To evaluate the impact of age on WMH, we performed a sensitivity analysis using a small sample matched for age and gender in CN and AD groups, alongside an examination of the interaction between age and diagnosis. The findings revealed that, after rigorously controlling for age and gender, the difference in WMH between the CN and AD groups was no longer statistically significant (*t* = –1.042, *P* = 0.328) (Table 3). Once the ages of the CN and AD groups were precisely matched, the disparity in WMH between the two groups was eliminated (*B* = –296.766, *P* = 0.759) (Table 3), suggesting that the influence of age on WMH is comparable in cognitively normal elderly individuals and those with AD.

**TABLE 3:** Summary of sensitivity and interaction analyses for WMH volume.

Analysis	CN	AD	Effect Size (95% CI)	*P*-value
Sensitivity analysis*				
WMH volume, mm^3^	9177.88 ± 14651.31	29468.90 ± 41021.88	Mean diff: –20291.01 (–65213.19, 24631.17)	0.328
Interaction analysis**				
Age effect (β per year)	35.477	–261.289	Interaction β = –296.766	0.759

*Age- and sex-matched subsample. Independent samples *t*-test.

^**^Linear regression with centered age, diagnosis, and age × diagnosis term, adjusted for sex.

### 3.4 HSPA8 protein combinated with WMH predicted the early AD

**FIGURE 8:**
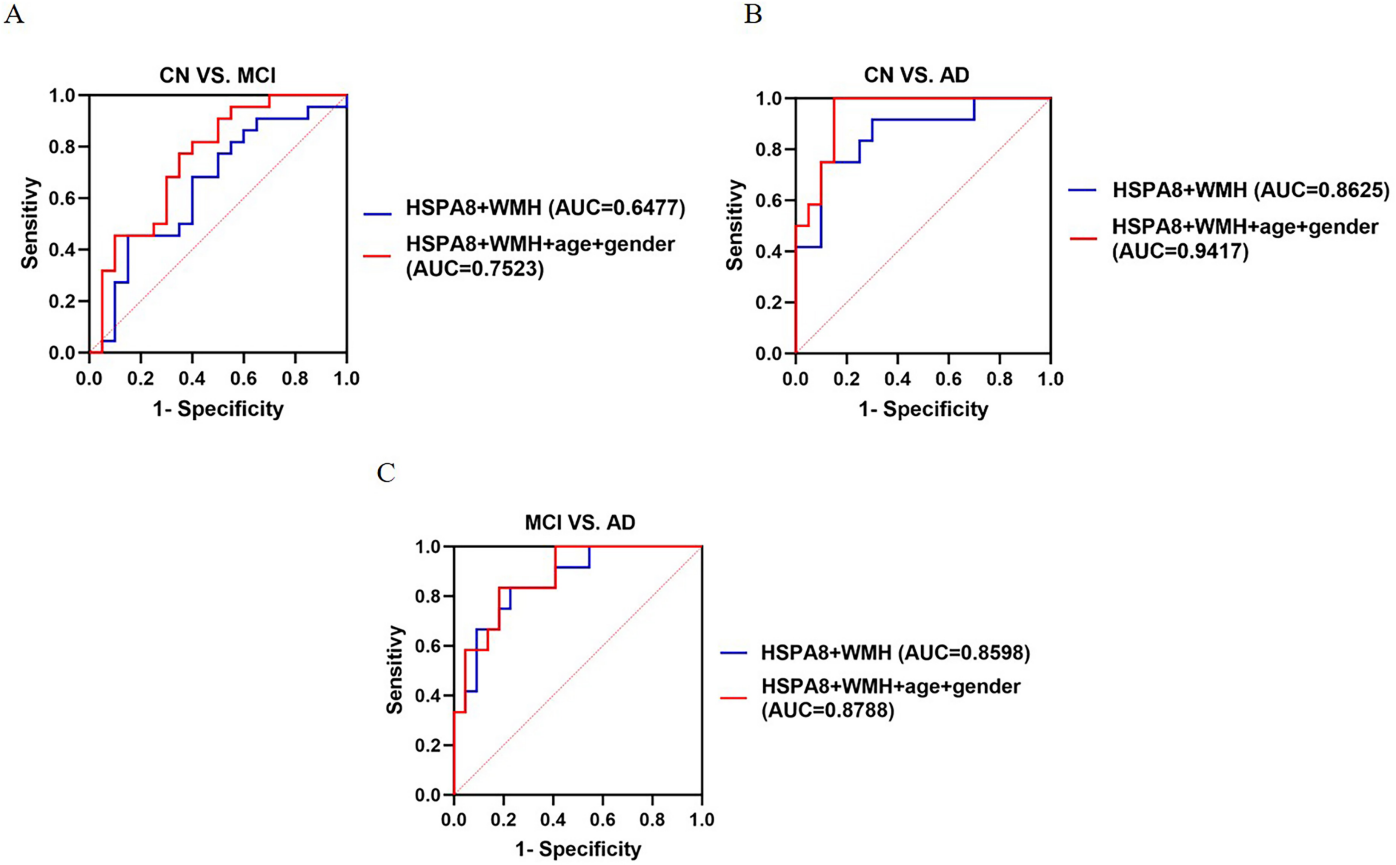
The AUC of HSPA8 combinated with WMH to the AD disease continuum. **(A)** The ROC curve of HSPA8 and WMH to CN vs. MCI. **(B)** The ROC curve of HSPA8 and WMH to CN vs. AD. **(C)** The ROC curve of HSPA8 and WMH to MCI vs. AD.

The results indicate that HSPA8 protein and WMH are potential predictors of AD stages. The AUC values improved when combined: from 0.5966 (HSPA8) and 0.5747 (WMH) to 0.6477 for distinguishing MCI from controls; from 0.6667 (HSPA8) and 0.8571 (WMH) to 0.8625 for AD from controls; and from 0.7255 (HSPA8) and 0.8455 (WMH) to 0.8598 for MCI from AD. Incorporating age and gender further enhanced the AUC values to 0.7523, 0.9417, and 0.8788 for MCI vs. controls, AD vs. controls, and MCI vs. AD, respectively, surpassing those of HSPA8 and WMH alone (Figure 8).

## 4 Discussion

Alzheimer’s disease has a long incubation period, complicating early diagnosis. Recently, efforts have focused on early detection through blood biomarkers, though traditional markers like Aβ40, Aβ42, p-tau181, p-tau217, and GFAP have limitations due to variability across populations and ages. New markers are needed for better early diagnosis, and combining these with neuroimaging can enhance diagnostic accuracy. Blood proteomics methods such as Base-SOMA, Olink PEA, mass spectrometry, and 4D-DIA can help discover new markers. Walker et al. (2023) identified 32 plasma proteins related to dementia risk (such as proteostasis, immunity, synaptic function, and extracellular matrix organization) using SOMAmer-based proteomics. Dark et al. (2024) found 14 protein health indices from SomaSignal^®^ tests that may impact Alzheimer’s and neurodegenerative diseases, particularly NfL levels. Mofrad et al. (2022) identified 12 plasma proteins that effectively distinguish FTD from AD (AUC: 0.99) using SomaScan Regression. Ehtewish et al. (2023) used PEA proteomics to identify dementia biomarkers, finding proteins like NEFL, IL17D, WNT9A, and PGF negatively linked to cognitive performance. Mehta et al. (2023) conducted a targeted mass spectrometry assay to measure 269 plasma proteins, identifying biomarkers linked to cognitive function in men without severe impairment. Inoue et al. (2023) used liquid chromatography-mass spectrometry to link plasma proteins to MCI and AD, focusing on coagulation, immunity, lipid metabolism, and nutrition. However, Few studies have used 4D-DIA proteomics to study the relationship between MCI, AD, and CN individuals.

We discovered HSPA8, IGFBP3, GPLD1, and Biotinidase were significantly down-regulated in the MCI/AD group. GO analysis indicated enrichment in the insulin-like growth factor receptor signaling pathway, while KEGG analysis highlighted the p53 and TNF signaling pathways, suggesting that aging and inflammation may play roles in AD progression. In this study, we utilized 4D-DIA proteomics to identify HSPA8, IGFBP3, GPLD1, and Biotinidase proteins in MCI vs. AD groups. HSPA8 exhibited superior AUC values compared to other proteins for distinguishing CN from MCI and MCI from AD. Our research identified a declining trend in the expression of HSPA8; however, this trend did not reach statistical significance within the AD continuum. This lack of significance may be attributed to missing values in the relative quantification of HSPA8 proteomics and the relatively concentrated nature of its quantification values. Additionally, WMH volume correlated with AD progression. Notably, combining HSPA8 and WMH improved early AD diagnostic accuracy, and including age and gender further increased the AUC value. These findings suggest that HSPA8 and WMH may serve as early AD biomarkers.

The HSP70 family plays a vital role in preventing Aβ plaque and tau aggregate formation (Lackie et al., 2017), with some studies indicating members of the heat shock protein family A (HSPA) as potential early AD biomarkers. The HSP70 family is composed by 17 members, HSPA8 is constitutively expressed among which (Campanella et al., 2018). Dong et al. (2022) highlighted HSPA1A, HSPA2, and HSPA8 as key immune-related genes in AD, suggesting their prognostic potential. Silva et al. (2014) found no significant difference in HSPA8 and HSPA9 mRNA expression in peripheral blood between elderly controls and AD patients, but observed significant downregulation in postmortem AD brain tissue. While previous research focused on HSPA gene expression, they missed the significance of HSPA8 protein in AD disease spectrum. Our study found HSPA8 protein had AUC values of 0.5966 for CN vs. MCI and 0.7255 for MCI vs. AD, surpassing IGFBP3, GPLD1, and Biotinidase. For CN vs. AD, the AUC values were 0.6667 for HSPA8, 0.7273 for IGFBP3, 0.6278 for GPLD1, and 0.6506 for Biotinidase, suggesting its potential as an early AD biomarker. We observed notable HSPA8 expression across the AD continuum and analyzed its relationship with cognitive scores (MMSE and MoCA) and WMH volume. However, due to a small sample size of MCI patients and missing data, the correlations were unclear.

The AAIC2024 report suggested incorporating vascular burden, like WMH, into the ATN framework to better study its relationship with AD. Research by Sargurupremraj et al. (2024) indicated a causal link between increased white matter signal burden and higher AD risk, while van den Berg et al. (2018) found a stronger association between WMHs and cognition in MCI patients than in AD patients by a meta-analysis. Some studies showed that AD and MCI patients have reduced white matter volume compared to normal controls (Radanovic et al., 2013). The WMH volumes in AD patients were assessed using FLAIR imaging with ITK-SNAP software in cases of intracerebral hematoma (Chen et al., 2018b), hemorrhage (Chen et al., 2018a), or stroke (Aamodt et al., 2021). We used ITK-SNAP software to assess WMH volumes in MCI, AD patients, and cognitively normal subjects. Our findings indicated that WMH volumes were higher in MCI and AD patients compared to cognitively normal individuals, with an increase correlating with the progression of AD. This aligns with previous studies, such as Wang et al. (2020) which found greater WMH volumes in AD patients than controls and MCI individuals, and Dadar et al. (2022) which reported significantly higher WMH volumes in all cognitively impaired groups compared to cognitively intact elderly individuals. Both our study and these reports support the notion that WMH may serve as a biomarker for the AD continuum.

To control for age-related confounding, we performed an age-matched sensitivity analysis and tested for age-diagnosis interaction. Results indicated no significant difference in WMH between CN and AD groups after age and sex matching (*P* > 0.05), and no significant age-diagnosis interaction (*P* > 0.05). This implies that WMH burden is primarily due to normal aging, with limited impact from AD pathology. The study highlights the importance of age-matched designs or strict age control in neurodegenerative research to prevent misinterpreting age-related changes as disease-specific markers.

The AUC values for the HSPA8 protein were 0.5966, 0.6667, and 0.7255 for MCI vs. CN, AD vs. CN, and MCI vs. AD, respectively. These findings suggest that the diagnostic efficacy of HSPA8 protein alone across the AD spectrum is relatively weak, indicating the necessity of combining HSPA8 with other biomarkers to enhance its diagnostic utility for AD. Importantly, combining blood protein analysis with neuroimaging improves early AD diagnostic accuracy, with AUC values for HSPA8 protein and WMH combinations reaching 0.6477, 0.8625, and 0.8598 for MCI/CN, AD/CN, and MCI/AD, respectively, outperforming each measure alone. Multivariable logistic regression analysis indicated significant differences in age and WMH between MCI and AD populations. Consequently, Age was added to the diagnostic model for WMH and HSPA8 protein, improving AUC values to 0.7523, 0.9417, and 0.8788 when combined with age and gender. Research by Shi et al. (2021) identified ApoB and TIM-1 proteins, linked to WMH in cognitively normal older adults using SOMAscan proteomics. Short et al. (2023) found no link between 1,305 plasma proteins and hippocampal or WMH volume in the Framingham Heart Study Offspring cohort. This suggests that combining WMH and HSPA8 proteins may partially enhance early AD diagnosis.

HSPA8, crucial in CMA (Endicott, 2024), may reduce neuroinflammation in neurodegenerative diseases (Wu et al., 2025), with evidence showing that its inhibition reduces spinal cord ischemia-reperfusion neuroinflammation (Mi et al., 2021). Garnier-Crussard et al. (2022) linked WMH to cortical AD pathology through Wallerian degeneration and inflammation. We propose that reduced HSPA8 protein could lead to WMH via neuroinflammation from impaired CMA, pending experimental validation.

This study has several limitations. First, the AUC value for the HSPA8 protein related to the AD spectrum is low, with modest and statistically insignificant correlation coefficients with the MMSE and MoCA, possibly due to focusing on a single protein or a small sample size. Second, this study lacks a correlation analysis between HSPA8 and the key pathological proteins Aβ or p-tau in AD, and while a correlation with WMH was noted, it lacked statistical significance. Third, combining HSPA8 and WMH may improve diagnostic performance for the AD spectrum, indicating that key CMA-related protein HSPA8 might partially mediate WMH development, though this requires further validation through clinical and animal studies. Finally, the findings need confirmation from an external validation cohort.

## 5 Conclusion

Our study indicates that the expression of the HSPA8 protein decreases across the AD spectrum, while WMH exhibit a significant upward trend. Age is the primary factor influencing WMH variation, exerting a similar effect in both CN and AD groups. AD disease status does not significantly impact WMH levels or the rate of WMH change with age. Consequently, age must be carefully considered when interpreting increased WMH in AD patients. To some extent, the combined assessment of HSPA8 and WMH may serve as early biomarkers for AD, thereby enhancing early detection. Incorporating age and gender into the diagnostic model improves the AUC value, underscoring the importance of HSPA8 and WMH in the early diagnosis of AD.

## Data Availability

The datasets presented in this study can be found in online repositories. The names of the repository/repositories and accession number(s) can be found in the article/Supplementary material.

## Supplementary material

Supplementary Table 1 The original data of plasma 4D-DIA proteomics.

Supplementary Table 2 The table of demographic, clinical characteristics and WMH volume datas of the study participants.
